# Multifunctional Sr,Mg-Doped Mesoporous Bioactive Glass Nanoparticles for Simultaneous Bone Regeneration and Drug Delivery

**DOI:** 10.3390/ijms25158066

**Published:** 2024-07-24

**Authors:** Tamara Matic, Farah Daou, Andrea Cochis, Nemanja Barac, Vukasin Ugrinovic, Lia Rimondini, Djordje Veljovic

**Affiliations:** 1 Faculty of Technology and Metallurgy, University of Belgrade, Karnegijeva 4, 11000 Belgrade, Serbia; tmatic@tmf.bg.ac.rs (T.M.); djveljovic@tmf.bg.ac.rs (D.V.); 2 Department of Health Sciences, Center for Translational Research on Autoimmune and Allergic Diseases (CAAD), Università del Piemonte Orientale (UPO), Corso Trieste 15A, 28100 Novara, Italy; farah.daou@uniupo.it (F.D.); andrea.cochis@med.uniupo.it (A.C.); 3 Innovation Center of the Faculty of Technology and Metallurgy Ltd., Karnegijeva 4, 11000 Belgrade, Serbia; nbarac@tmf.bg.ac.rs (N.B.); vugrinovic@tmf.bg.ac.rs (V.U.)

**Keywords:** mesoporosity, bioactive glass, 3D cell culture model, EA.hy926, BM-MSC, ion-doping

## Abstract

Mesoporous bioactive glass nanoparticles (MBGNs) doped with therapeutical ions present multifunctional systems that enable a synergistic outcome through the dual delivery of drugs and ions. The aim of this study was to evaluate influence of co-doping with strontium and magnesium ions (SrMg-MBGNs) on the properties of MBGNs. A modified microemulsion-assisted sol–gel synthesis was used to obtain particles, and their physicochemical properties, bioactivity, and drug-loading/release ability were evaluated. Indirect biological assays using 2D and 3D cell culture models on human bone marrow-derived mesenchymal stem cells (hBM-MSCs) and endothelial EA.hy926 cells, respectively, were used to determine biocompatibility of MBGNs, their influence on alkaline phosphatase (ALP) production, calcium deposition, and cytoskeletal organization. Results showed that Sr,Mg-doping increased pore volume and solubility, and changed the mesoporous structure from worm-like to radial–dendritic, which led to a slightly accelerated drug release compared to pristine MBGNs. Biological assays confirmed that particles are biocompatible, and have ability to slightly induce ALP production and calcium deposition of hBM-MSCs, as well as to significantly improve the proliferation of EA.hy926 compared to biochemical stimulation via vascular endothelial growth factor (VEGF) administration or regular media. Fluorescence staining revealed that SrMg-MBGNs had a similar effect on EA.hy926 cytoskeletal organization to the VEGF group. In conclusion, Sr,Mg-MBGNs might be considered promising biomaterial for biomedical applications.

## 1. Introduction

Bioactive glass (BG) particles have been broadly investigated and applied as a biomaterial for hard tissue repair and regeneration ever since the Hench’s discovery of 45S5 Bioglass^®^ in 1969 [1]. In the 1990s, sol–gel BGs obtained at low temperatures emerged as “second generation BGs” with higher specific surface area and improved bioactivity [2,3]. However, their application was intended for implantation only, since porosity was insufficient for them to be used as drug carriers. Since 2001, mesoporous silica-based materials have been investigated as a drug delivery system [4,5,6], nonetheless they lacked bioactivity. The development of the so-called “third generation” mesoporous bioactive glass particles (MBGs) in 2004 presented a major breakthrough in the field of biomaterials as it combined sol–gel technology with supramolecular chemistry [7]. The mesoporous channels in MBGs improve textural properties and bioactivity compared to sol–gel BGs, while their large pore volume allows simultaneous loading of drugs, growth factors, or other bioactive molecules [8]. Additionally, the silanol groups at the surface of MBGs enable further modification strategies with different functional groups for controlled release of therapeutic compounds [9,10].

Recently, mesoporous bioactive glass nanoparticles (MBGNs) doped with therapeutical ions have emerged as a new concept of multifunctional systems with dual delivery of drugs and ions, which provides synergistic outcomes [8,9]. MBGNs are characterized by a particle size ranging from 20 to 800 nm and mesopores ranging from 2 to 50 nm [9]. Numerous research groups worldwide have been investigating the incorporation of an extensive variety of ions, including Li^+^, Ag^+^, Sr^2+^, Cu^2+^, Co^3+^, Zn^2+^, Mg^2+^, Mn^2+^, Ce^4+^, Ga^3+^, Te^4+^, Se^4+^, etc. [11,12,13,14,15,16,17,18,19,20], into the MBGN structure to achieve optimal physicochemical and textural properties, in addition to specific therapeutic effects, such as osteogenic, antibacterial, angiogenic, anti-inflammatory, antitumor, hemostatic, etc., which are induced by the release of the ions. The excellent structural characteristics, easy surface modification, great bioactivity, biocompatibility, and osteoconductivity, as well as the capacity to locally deliver drugs and ions, make ion-doped MBGNs a unique and versatile multitherapy system. As such, they have a potential application in orthopedic surgery and dentistry, including bone grafting materials, cements, implant coatings, bioactive fillers in dental composites, in addition to soft and hard tissue engineering, drug/growth factor delivery systems, and cancer diagnosis and therapy [10,21,22,23].

There are over 150 million new bone fractures worldwide every year caused by trauma, infection, or tumor treatment [24]. In critical-size bone defects, a nonunion is formed and bone is unable to heal spontaneously; thus, bone substitutes are used to bridge the gap and promote bone healing [25,26]. Since bone, especially cancellous bone, is a highly vascularized tissue, a successful bone regeneration approach needs to focus not only on promoting new bone formation, but also on the formation of new blood vessels. Ionic dissolution products from silica-based materials were shown to significantly enhance the angiogenic capability of endothelial cells [27]. Strontium (Sr^2+^) is well known to stimulate osteogenesis in both healthy and osteoporotic bone by having a dual role: stimulation of osteoblast activity and suppression of osteoclast activity [28]. Sr^2+^ ions were found to promote osteogenic differentiation of mesenchymal stem cells (MSC) in vitro by upregulating the expression of osteoblast marker genes (collagen type I, osteocalcin, and osteopontin), as well as increasing alkaline phosphatase (ALP) activity and matrix mineralization [29,30,31]. Magnesium (Mg^2+^) is one of the most abundant elements in human body, mainly stored in bone tissue, and plays an important role in the bone remodeling process. Mg^2+^ ions were shown to stimulate both osteogenesis and angiogenesis, with enhanced cell proliferation, differentiation, and matrix mineralization in human bone marrow-derived MSCs (BM-MSCs) and osteoblasts in vitro, and also with the stimulation of vascular endothelial growth factor (VEGF) production and neovascularization [29,32,33,34,35,36].

Although incorporation of Sr^2+^ and Mg^2+^ ions as single-ion dopants in MBGs was the focus of many studies [11,15,37,38,39,40,41] there are still limited reports on their joint effect [42]. Co-doping of MBGNs with Sr^2+^ and Mg^2+^ ions might lead to accelerated bone regeneration. However, it can also influence the solubility and textural properties of MBGNs, and hence, affect bioactivity [11,43] and drug loading and release [44]. Since bone-related pathologies are often related to inflammatory processes associated with infections, the loading of anti-inflammatory agents on MBGNs, such as ibuprofen and diclofenac, and their local release at the implementation site has recently gained great interest for improved bone regeneration [22].

The objective of this study was to obtain multifunctional MBGNs binary doped with Sr^2+^ and Mg^2+^ ions for stimulated bone regeneration with the ability to deliver drugs. This study aimed to investigate the Sr,Mg-MBGNs ability to load and release the nonsteroidal, anti-inflammatory drug ibuprofen. Additionally, it investigated the influence of Sr,Mg-doping on the physicochemical and textural properties, as well as pro-osteogenic and pro-angiogenic properties in 2D and 3D cell culture models, on human bone marrow-derived mesenchymal stem cells (hBM-MSCs) and endothelial EA.hy926 cells, respectively. This study shows that simultaneous doping with Sr and Mg ions leads to increased pore volume and solubility of the particles, but also affects their mesoporosity by transforming the mesoporous structure from worm-like to radial–dendritic. This modification affects drug-loading capacity, and results in slightly accelerated drug release of SrMg-doped particles compared to pristine MBGNs. For anti-inflammatory drugs, such as ibuprofen, accelerated release could provide a quicker pain and/or inflammation relief, which is beneficial for acute conditions such as post-surgical recovery. The indirect biological evaluation assays confirmed that both pristine and doped particles are biocompatible and capable of modestly enhancing alkaline phosphatase (ALP) production and calcium deposition in hBM-MSCs. Both particle types significantly boosted the proliferation of EA.hy926 cells when compared to biochemical stimulation via vascular endothelial growth factor (VEGF) administration or by using regular media. Moreover, fluorescence staining revealed that SrMg-MBGNs had a similar effect on the cytoskeletal organization of EA.hy926 cells as the VEGF group in 3D cell culture, suggesting a pro-angiogenic effect of the binary doped Sr,Mg-MBGNs.

## 2. Results and Discussion

### 2.1. Compositional Analysis

**Figure 1:**
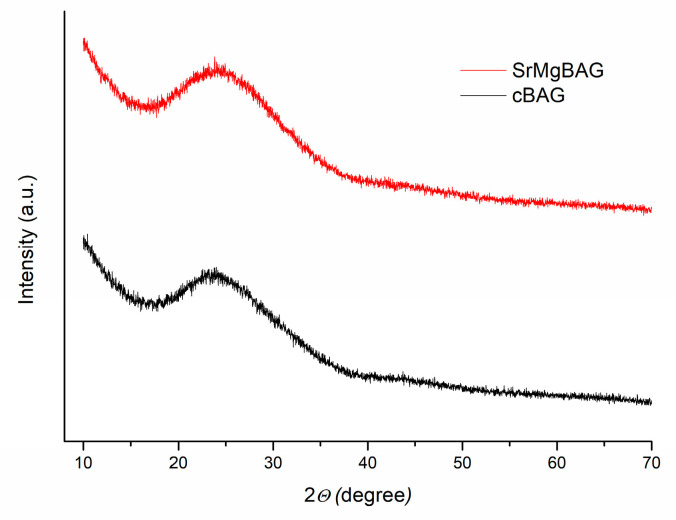
XRD diffractogram of pure (cBAG) and Sr,Mg-doped (SrMgBAG) MBGNs.

Sr,Mg-doped MBGNs were successfully developed by a microemulsion synthesis route combined with high-frequency ultrasonication. The X-ray diffraction (XRD) analysis (Figure 1) revealed a broad halo in the 2*Θ* range of 18–30° confirming the amorphous nature of the particles. No diffraction peaks of crystalline phases such as MgO, SrO, and CaO upon calcination at 600 °C were observed, indicating that dopants were fully incorporated in the glassy structure of the MBGNs.

Table 1 shows the compositions of the obtained particles determined by inductively coupled plasma mass spectrometry (ICP), and a discrepancy with nominal composition of glasses is observed, as expected. The main drawback of the microemulsion technique is the lack of composition control. In base-catalyzed sol–gel synthesis, metal ion salts are added upon formation of silica nanoparticles; hence, metallic ions are only loosely adsorbed on the silica surface until they are incorporated in the glass structure during the calcination process [23,45]. As a result, the ions are easily washed off during excessive rinsing, which is necessary to eliminate the organic phase and avoid agglomeration.

**Figure 2:**
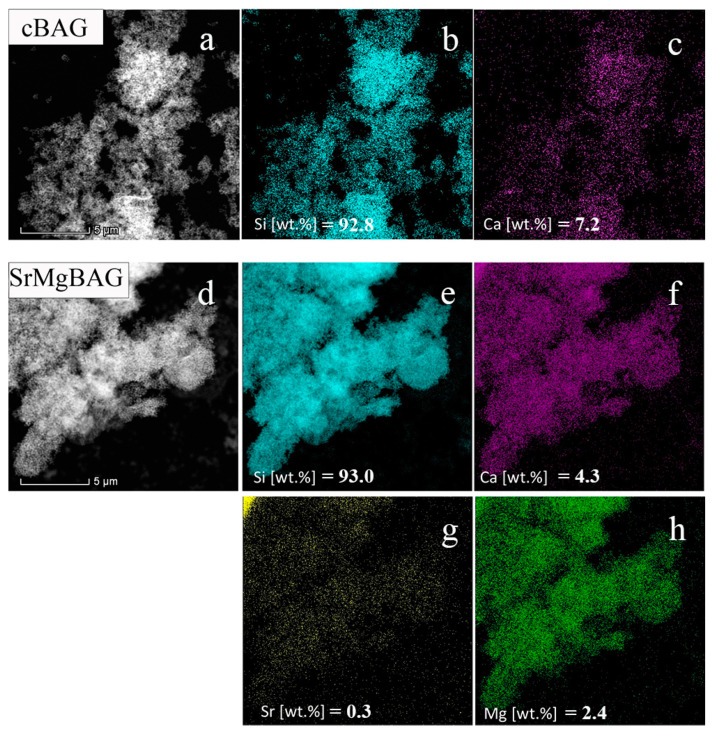
EDX elemental mapping results of the analyzed regions (black/white figures) related to pristine cBAG (**a**–**c**) and doped Sr,MgBAG powder (**d**–**h**).

Interestingly, in this study, the co-doping of the same nominal composition of Mg^2+^ and Sr^2+^ in MBGNs resulted in a much lower content of SrO than MgO (0.26 vs. 4.74 mol.%). This could suggest easier adsorption of the more electronegative Mg^2+^ ions compared to Sr^2+^ and Ca^2+^. Total content of SrO + MgO was 5 mol.%, and energy-dispersive X-ray spectroscopy (EDX) mapping coupled with transmission electron microscopy (TEM) (displayed in Figure 2) showed a homogeneous distribution of elements throughout the particles, with Si most prominent, which is in accordance with the composition of the glasses.

### 2.2. Morphology and Textural Properties 

Simultaneous incorporation of Sr^2+^ and Mg^2+^ ions into MBGNs did not affect the specific surface area (SSA) compared to pristine cBAG, but increased the total pore volume (0.92 vs. 0.84 cm^3^/g) and the average pore size (9.5 vs. 7.4 nm). Compared to microemulsion synthesis of pristine MBG by a similar method [43], the simultaneous application of ultrasound led to a decrease in CaO content, as well as an increase in SSA from 229 to 386 m^2^/g and an increase in total pore volume from 0.29 to 0.84 cm^3^/g (Table 2). On the other hand, the average particle size was not affected. Particle size of cBAG ranged from 67 to 164 nm, with an average of 118 nm, while the particle size of SrMgBAG was found to be in the range 70 to 156 nm with an average of 112 nm.

As Liang et al. previously showed, the particle size of spherical MBG can be altered between 130 and 250 nm by changing the ammonia concentration during microemulsion synthesis, which accordingly influences their SSA to be in range of 685 to 259 m^2^/g [46]. Sr,Mg-doping increased ζ—potential compared to cBAG, which was more noticeable in ethanol than in water (Table 2). This could suggest easier adsorption of electropositive ions and molecules on the surface of SrMgBAG compared to cBAG.

**Figure 3:**
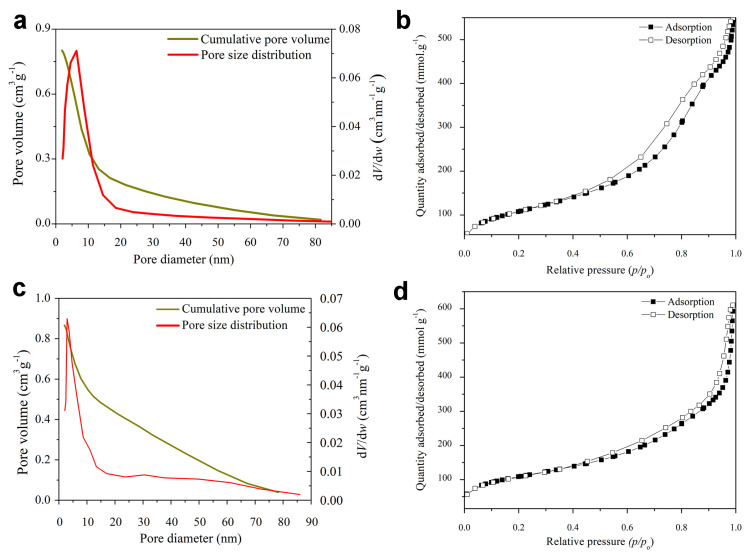
Pore size distribution and adsorption/desorption curves of bioactive glass particles for cBAG (**a**,**b**) and SrMgBAG (**c**,**d**).

The adsorption/desorption curves, represented in Figure 3, depict a type IV isotherm according to the International Union of Pure and Applied Chemistry (IUPAC), with a type 3 hysteresis loop characteristic of mesoporous materials with slit-shaped pores. A somewhat thinner hysteresis loop is visible for SrMgBAG sample, which corresponds to the smaller pore size and narrower pore size distribution.

**Figure 4:**
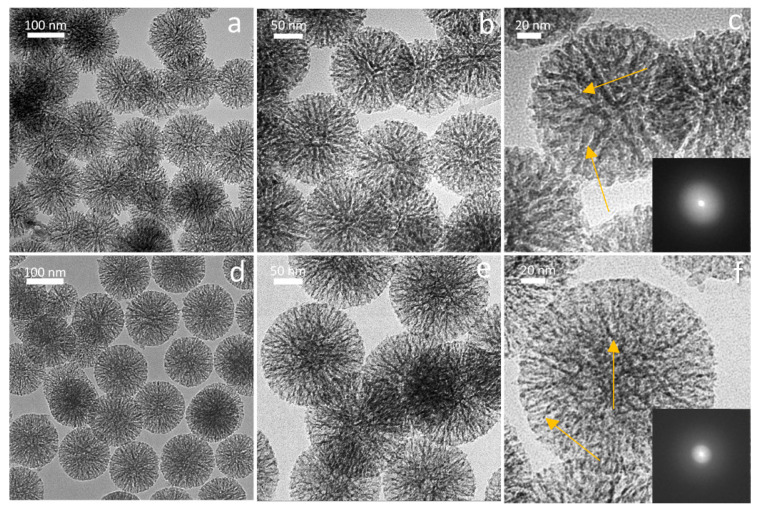
TEM micrographs and fast Fourier transform (FFT) image of the pristine cBAG mesoporous bioactive glass particles (**a**–**c**, worm-like mesostructures are indicated by the yellow arrow), and Sr,Mg-doped SrMgBAG (**d**–**f**, dendritic mesoporous channels are indicated by the yellow arrow).

The addition of positively charged metallic ions during microemulsion synthesis can lead to electrostatic interaction with a pore-forming cationic surfactant, such as Cetyltrimethylammonium bromide (CTAB), resulting in affected textural properties and disordered worm-like mesopore structure [23,47]. TEM micrographs of the cBAG particles’ cross-section showed worm-like mesostructure (Figure 4c, indicated by the yellow arrow), while in SrMgBAG radial dendritic mesoporous channels were found (Figure 4f, indicated by the yellow arrow). These structural differences could be a result of a higher number of cations present in the system and their interaction with CTAB, but also influenced by the increased stirring time during the microemulsion synthesis of SrMgBAG compared to cBAG [48,49,50].

Average pore diameter in SrMgBAG was found to be larger than in cBAG (9.53 nm vs. 7.56 nm, respectively). However, this is a result of the bimodal pore size distribution of the radial dendritic mesopores in SrMgBAG, being larger at the outer particle surface, and smaller inside the particle (Figure 3 and Figure 4). Despite the most abundant pores in SrMgBAG being much smaller (2.92 nm) than in cBAG (6.38 nm), both showed to have pores in the range 2–10 nm, which is considered optimal for hosting and releasing drugs and other biological molecules [44]. Fast Fourier transform (FFT) images of the obtained particles confirmed the amorphous nature of undoped and doped particles, which was in agreement with the diffractograms shown in Figure 1.

### 2.3. In Vitro Bioactivity

Bioactivity of a material presents its ability to grow new crystals of biomimetic hydroxyapatite (usually hydroxy-carbonate apatite—HCA) on its surface upon immersion in body-simulated fluid (SBF). The ion-doping of glasses influences their solubility and hence can affect the bioactivity [43,44]. It was previously shown that the addition of Mg^2+^ ions in MBGNs in the SiO_2_-CaO system leads to a retarding effect on bioactivity, specifically on the crystallization of the amorphous Ca-P layer [11]. Even after 14 days of immersion in SBF, no HCA formation was visible when more than 3 mol.% of MgO was incorporated. On the other hand, Sr^2+^ substitution was not found to have opposing effect on the bioactivity of glasses [39,51].

**Figure 5:**
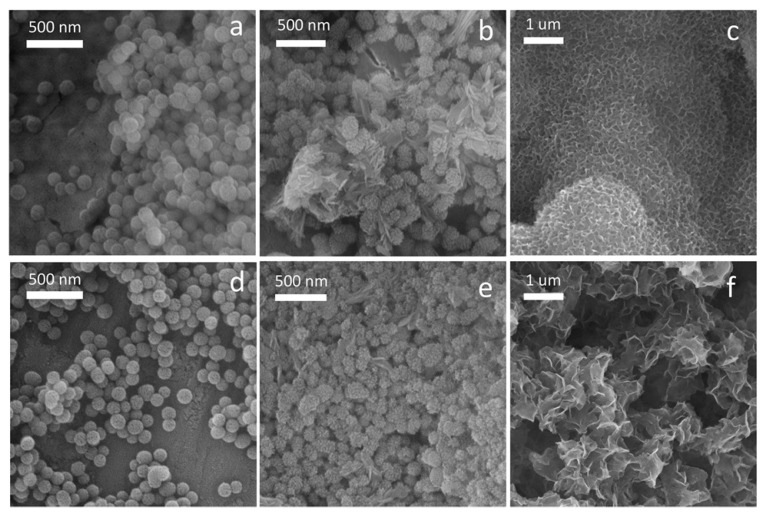
Bioactivity test: pristine (cBAG) (**a**–**c**) and Sr,Mg-doped (SrMgBAG) particles (**d**–**f**) before and after soaking in SBF for 7 days (**b**,**e**) and 14 days (**c**,**f**).

In the present study, MBGNs containing 4.76 mol.% MgO and 0.24 mol.% SrO were shown to be bioactive with the formation of HCA crystallites upon immersion in SBF (Figure 5). After 7 days, the surface of cBAG and SrMgBAG had a rough appearance due to dissolution of glass and precipitation of nanocrystals on the surface, accompanied by formation of needle-like crystals characteristic of HCA (Figure 5b,e). After 14 days of immersion in SBF, the surface of both cBAG and SrMgBAG was completely covered in a thick layer of the apatite crystals, thus confirming the bioactive nature of the MBGNs (Figure 5c,f).

### 2.4. Dissolution and Ion Release Evaluation

**Figure 6:**
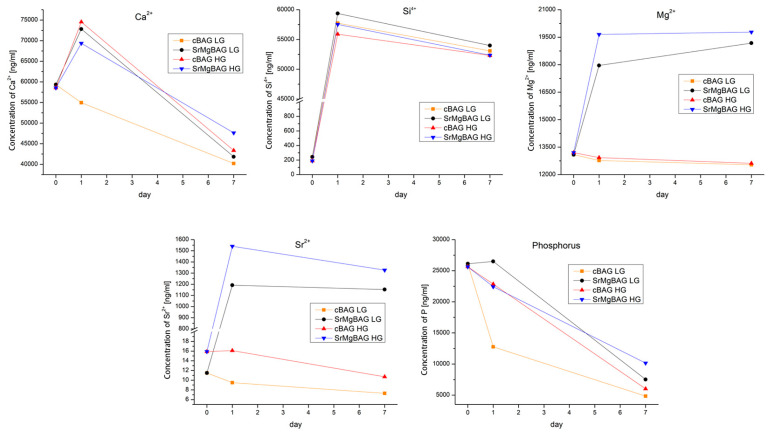
Ion release profiles of bioactive glass particles of control glass (cBAG) and SrMg-doped glass (SrMgBAG) in two DMEM culture media: high glucose (HG) and low glucose (LG).

The vitreous network of silica is formed via Si-O-Si bridging oxygen atoms. The introduction of modifier cations (such as Ca^2+^, Sr^2+^, Mg^2+^) into the glass disrupts the network connectivity by formation of the nonbridging oxygen atoms, which improves the dissolution behavior. However, MgO is an intermediate oxide and can also penetrate into the backbone of the glass structure acting as a network former, depending on the MgO content [52]. Data from the literature concerning the effects of Mg incorporation on the bioactive glass dissolution show a retarding effect for both the melt-quenched and sol–gel glasses [53,54,55]**.** On the contrary, in the case of Mg-doped MBGNs, the addition of Mg^2+^ showed that increase in Mg^2+^ content led to a higher dissolution rate, which was assigned to the increase in SSA [11]. In the present study, Sr,Mg doping of MBGNs led to a slightly increased dissolution rate that was revealed by a higher concentration of silicon released in both high-glucose (HG) and low-glucose (LG) types of regular cell culture media (DMEM) compared to pristine cBAG (59 vs. 57 ppm, Figure 6), despite their similar SSA. This is in accordance with finding that a decrease in SiO_2_ content in MBGs leads to decreased resistance to degradation [56].

Inductively coupled plasma optical emission spectroscopy (ICP-OES) results for the concentration of dissolution products of the bioactive glass particles immersed in cell culture media are shown in the Figure 6. The ion-release profiles were examined in two types of regular cell culture media: DMEM low glucose (LG) and DMEM high glucose (HG). There was a sharp increase in silicon in both culture media after 24 h, but a higher concentration was found in DMEM-LG, indicating better solubility of glasses than in DMEM-HG culture media. The serum proteins in culture media can adsorb at the surface of particles forming protein corona, which can influence reactivity of the material [57]; hence, different dissolution behavior in the two cell culture media types was expected.

In the case of calcium, there was an increase in concentration in all samples after 24 h, except for cBAG in DMEM-LG. Higher Ca^2+^ content released in DMEM-HG from cBAG than SrMgBAG is in accordance with their chemical composition. In the case of SrMgBAG, as expected, there was an increase in the concentration of Sr^2+^ and Mg^2+^ ions in the culture media after 24 h, but in this case a greater concentration was found in DMEM-HG. A decrease in calcium and strontium content in the media was observed after 7 days. This is associated with the precipitation of the inorganic salts that were eliminated during the filtration process, which is supported by the decrease in phosphorus content.

The binary-doped Sr,Mg-MBGNs showed release of therapeutical ions after 24 h, ranging from 4.5 to 6 ppm of Mg^2+^ and 1.2 to 1.6 ppm of Sr^2+^, depending on the DMEM type. Previously, it was found that the concentration of Sr^2+^ ions in the range of 3 to 6 ppm released from Sr-BGs stimulate osteogenic response in hMSCs [30,31]. Total concentration of Mg^2+^ ions found in SrMg-BAG extracts were 18–19.5 ppm, which is considered to be a physiological concentration of Mg^2+^ (0.8 mM). The addition of 0.8–10 mM of Mg^2+^ (about 19–240 ppm) has been previously investigated in MSCs to determine its effect on osteogenesis. Yoshizawa et al. demonstrated that the addition of 10 mM of MgSO_4_ led to faster proliferation of hBMSc and increased osteogenic gene expression, matrix production, and mineral deposition [34], while Diaz-Tocados et al. found that increasing MgCl_2_ concentration from 0.8 to 1.8 mM enhanced proliferation by Notch1 signaling activation and induced osteogenic differentiation [58].

### 2.5. Assessment of Biological Properties

**Figure 7:**
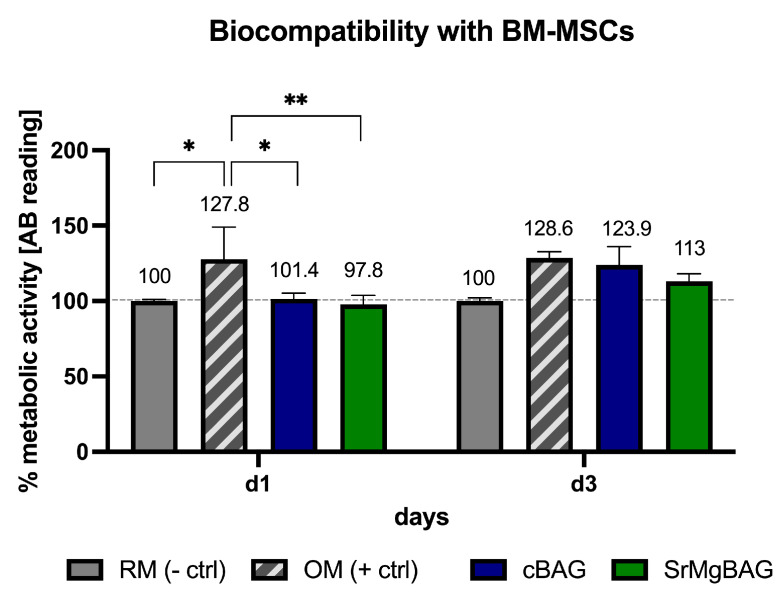
Metabolic activity of BM-MSCs in the 2D cell culture model after 1 and 3 days of culturing in regular media (RM), osteogenic media (OM), and media conditioned with 1 mg/mL cBAG or SrMgBAG [* *p* < 0.05, ** *p* < 0.01]. Bars represent means ± SD of *n* = 3 biological replicates.

The biocompatibility of the ionic dissolution products of the MBGN powders to BM-MSCs was indirectly evaluated using a resazurin assay. As shown in Figure 7, the ionic dissolution products of cBAG and SrMgBAG powders were not cytotoxic to BM-MSCs at a concentration of 1 mg/mL (the concentration used often ranges from 0.001 to 1 mg/mL [30]). After 24 h of culturing the BM-MSCs, the group with osteogenic medium showed a significant increase in metabolic activity compared to RM (*p* = 0.015), cBAG (*p* = 0.029), and SrMgBAG (*p* = 0.006); however, after 3 days no significant difference in BM-MSCs metabolic activity among the groups was observed. These results are in line with previous studies that showed no impairment in the cytocompatibility of MBGN following the addition of either Sr^2+^ or Mg^2+^ ions [41,59,60,61] or simultaneous Sr^2+^ and Mg^2+^ doping of MBGN [42].

**Figure 8:**
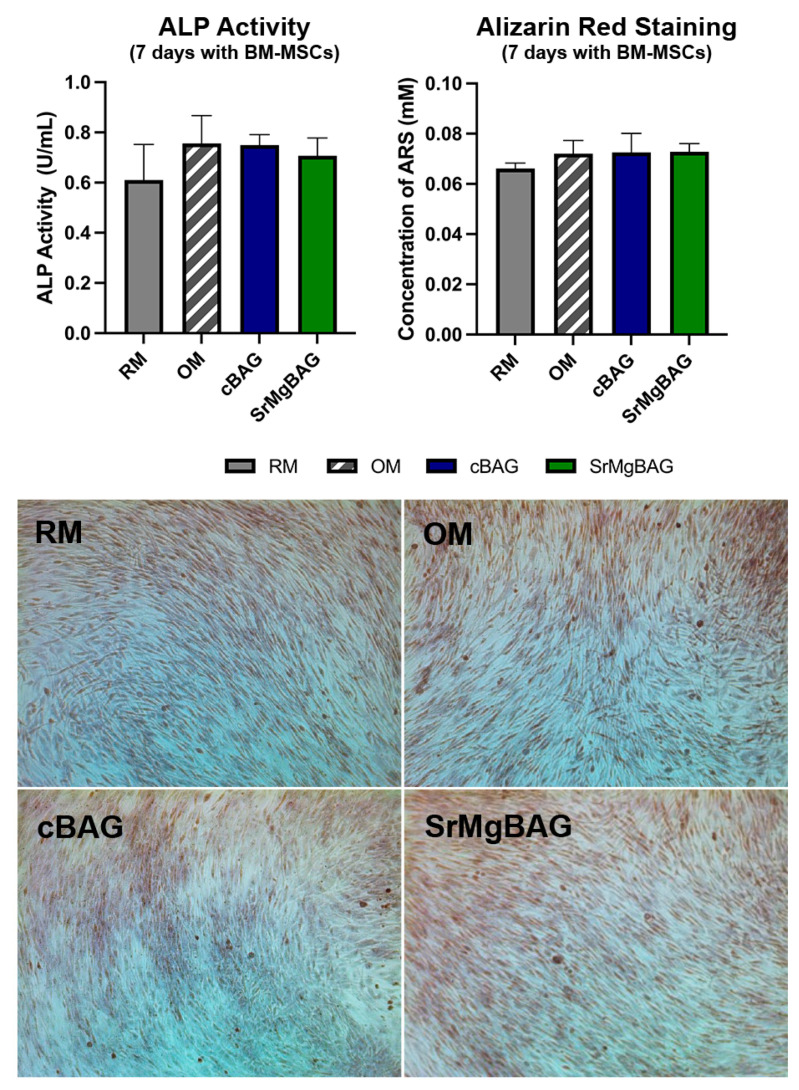
Alkaline phosphatase (ALP) activity and Alizarin Red staining of bone-like calcium deposits after 7 days of culturing with BM-MSCs; RM-regular media, OM-osteogenic media, and media conditioned for 24 h with 1 mg/mL of cBAG or SrMgBAG. Bars represent means ± SD of *n* = 3 biological replicates.

Alkaline phosphatase (ALP) activity is a widely used early marker of osteogenic differentiation in vitro, with levels increasing after 5 to 14 days of incubation. On the other hand, extracellular matrix (ECM) calcification that occurs during the late stage of osteogenic differentiation is identified by Alizarin Red (ARS) staining of calcium deposits [16]. After 7 days of culturing, a slight increase in ALP activity was found in the BM-MSCs cultured in osteogenic medium (OM), as well as in the presence of the released ions from the cBAG and SrMgBAG powders (Figure 8). This increase, however, was not statistically significant (*p*-value > 0.05) for the three groups in comparison to the control group. Qualitative ARS staining images showed higher calcium deposition in the presence of the released ions from the cBAG and SrMgBAG powders, and in the presence of OM (Figure 8). Quantitative ARS staining findings were consistent with qualitative results; however, the differences in ARS concentrations in the test samples were not significantly higher than those of the control group (*p*-value > 0.05).

There was no significant difference found between the SrMgBAG and cBAG effects on BM-MSCs, which was probably caused by the low concentrations of Sr^2+^ and Mg^2+^ ions released from the MBGNs compared to the previous studies [30,31,32], as revealed by the ICP analysis (Figure 6). Both SrMgBAG and cBAG particles were found to be able to slightly induce the osteogenic potential of human BM-MSCs due to the improvement in ALP activity and calcium deposition during the early stage. The absence of significant induction of osteogenesis found can be due to the short cultivation period (7 days) that was insufficient to cause a promotion in ALP activity or calcium deposition, which is in accordance with previous findings for Sr-BGNs, where significant mineralization was found after two weeks of culturing [31].

**Figure 9:**
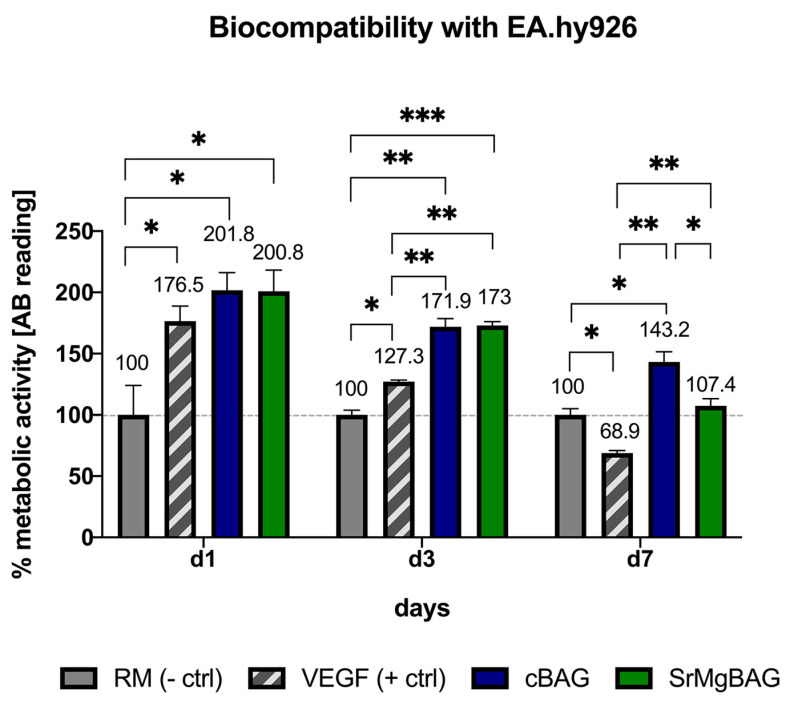
Metabolic activity of EA.hy926 endothelial cells in a 3D cell culture model after 1, 3, and 7 days of culturing in regular media (RM), media with 100 ng/mL vascular endothelial growth factor supplement (VEGF), and media conditioned with 1 mg/mL cBAG or SrMgBAG; [* *p* < 0.05, ** *p* < 0.01, *** *p* < 0.001]. Bars represent means ± SD of *n* = 3 biological replicates.

The biocompatibility of the ionic dissolution products of the MBGN powders to EA.hy926 cells was also indirectly evaluated using a resazurin assay (Figure 9). The ionic dissolution products of cBAG and SrMgBAG particles were also found to be cytocompatible with EA.hy926 cells. After only 24 h of culturing, ionic dissolution products of both types of glasses were shown to significantly increase EA.hy926 cell metabolic activity compared to the regular media (*p* = 0.010 for cBAG, *p* = 0.019 for SrMgBAG), similar to the VEGF effect. Given the remarkable increase in metabolic activity by over 75%, it indicates a substantial stimulation of EA.hy926 cell proliferation within the VEGF, cBAG, and SrMgBAG groups after just one day of culturing.

Interestingly, after 3 days of culturing both MBGN types were shown to have a significant effect on EA.hy926 cell metabolic activity compared to both RM (*p* = 0.001 for both cBAG and SrMgBAG) and VEGF (*p* = 0.007 for cBAG, *p* = 0.005 for SrMgBAG) (Figure 9). Since no significant difference was found among the MBGNs, it can be deducted that the angiogenic silicon ions play a crucial role in the stimulation of EA.hy926 cell metabolism and proliferation, which is in accordance with previous findings [27]. On the other hand, Sr^2+^ and Mg^2+^ doping did not show a significant effect on endothelial cells compared to cBAG due to their rather low concentration found in the extracts.

After 7 days of culturing, the metabolic activity was still higher in the presence of the ions released from the cBAG and SrMgBAG powders than the RM and VEGF groups; however, cBAG now showed a significantly higher metabolic activity (*p* = 0.027) compared to the SrMgBAG powder. On the other hand, the VEGF group showed a significant decrease in the metabolic activity of EA.hy926 on day 7 (*p* = 0.002 for cBAG, and *p* = 0.02 for SrMgBAG). Thus, the results show once again that the concentration of Sr^2+^ and Mg^2+^ ions released were not cytotoxic for EA.hy926 cells, while having a stimulating effect on the metabolic activity of endothelial cells.

**Figure 10:**
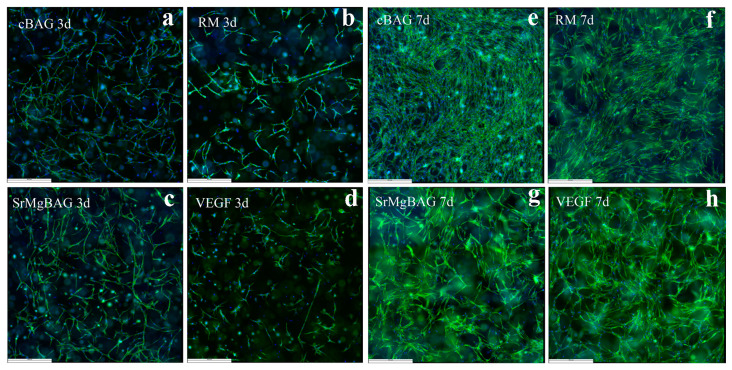
EA.hy926 endothelial cells in a 3D cell culture model after 3 and 7 days culturing with DMEM HG conditioned for 24 h with 1 mg/mL cBAG (**a**,**e**); DMEM HG (neg. ctrl, **b**,**f**); DMEM HG conditioned with 1 mg/mL SrMgBAG (**c**,**g**); and DMEM HG with 100 ng/mL VEGF (**d**,**h**). Cytoskeleton filaments are stained in green by phalloidin, while nuclei are stained in blue by DAPI. Scale bar: 450 μm, magnification 5×.

To examine the pro-angiogenic character of the particles, the cytoskeletal organization of the EA.hy926 cells was evaluated by fluorescence double-staining with phalloidin (actin f-filaments of the cytoskeleton) and 4′,6-diamidin-2-fenilindolo (DAPI, to visualize nuclei) after 3 and 7 days of culturing. As depicted in Figure 10, EA.hy926 cells exhibited well-formed elongated cytoskeletal organization. Overlapping growths were observed after 7 days of cultivation and were more prominent for EA.hy926 cells cultured in RM and with the released ions of cBAG. The latter can be attributed to a possible alteration in cell behavior due to the presence of VEGF and angiogenic Mg^2+^ ions released from the SrMg-BAG particles.

### 2.6. Drug Loading and Release

**Figure 11:**
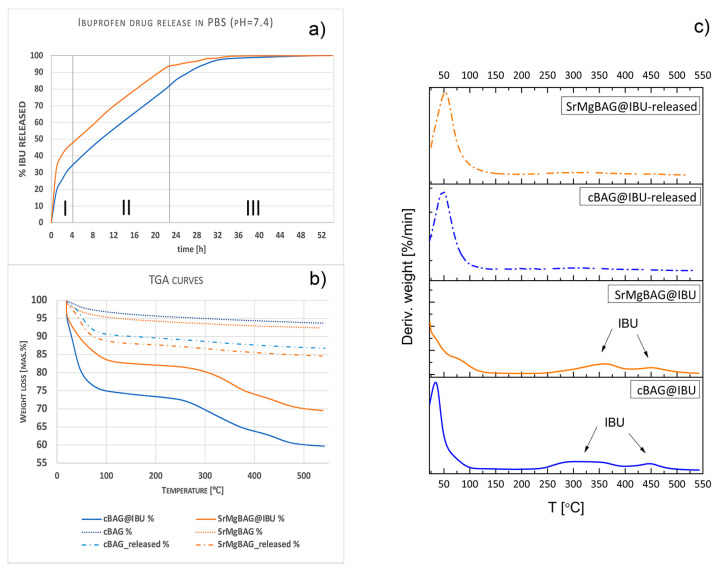
(**a**) Drug release profiles in 40 mL PBS (pH = 7.4) from mesoporous bioactive glass particles undoped (cBAG) and Sr,Mg-doped (SrMgBAG) loaded with IBU; (**b**,**c**) TGA-DTG curves of particles before and after loading with IBU, and after drug release.

MBGs can easily host drugs through adsorption at the surface, entrapment within the mesopores via covalent or noncovalent binding, or accommodation at the window of the MBGs [10]. The OH-rich surface gives MBGNs a negative surface charge which reduces its ability to load negatively charged drugs such as ibuprofen (IBU) [62]. Incorporation of metal oxides such as SrO or MgO in the silica network was previously shown to increase the loading capacity of IBU [9] owing to the high affinity between the alkaline functional groups and the acidic carboxyl groups of IBU. Additionally, a recent study reported that increasing the concentration of SrO from 0.02 to 1 mol.% in hydrothermally obtained BGs led to higher loading of IBU and more prolonged drug release [51]. However, in this study, the capacity of IBU drug loading was shown to slightly decrease upon incorporation of SrO and MgO from 15 mas. % for pristine to 12 mas. % for SrMgBAG (Figure 11), despite the total pore volume and ζ–potential being greater in the latter. The possible reason could relate to the much smaller pore size of the most abundant pores found in SrMgBAG (Table 2). Thermal analysis showed elimination of solvents up to 200 °C, and further decomposition of IBU taking place in two stages: (I) 220–400 °C and (II) 400–500 °C. After 55 h of drug-release in phosphate-buffered saline solution (PBS, pH = 7.4), the entire drug content was released, as shown by differential thermal gravimetric analysis (TGA/DTG) curves indicating no weight loss corresponding to IBU (Figure 11c).

Ion-doping in MBGs can influence solubility in the physiological medium and hence lead to modified drug-release kinetics [44]. In this study, Sr,Mg-doping of MBGNs was found to result in slightly faster IBU release compared to pristine MBGNs (Figure 11a). The drug release is generally promoted more from the radial dendritic mesoporous channels compared to the worm-like pores, due to being widely opened toward the external surface of the particle, allowing enhanced drug diffusion [49].

The drug release profiles obtained are characteristic of the MBGs having three steps [63]: the initial burst release (step I) of the physically adsorbed drug in the first 6 h, where 40 and 52% of IBU was released in cBAG and SrMgBAG, respectively, followed by a gradual diffusion of the drug entrapped in mesopores, up to 24 h (step II), before slowing and reaching a plateau (step III). Although having an initial burst release, when compared to the IBU released from mesoporous silica where drug release was completed after only 20 min [64], the drug release was much more sustained in cBAG and SrMgBAG. Further surface modification strategies as well as optimization of the drug-loading process (solvent type, loading duration, drug concentration, etc.) can be additionally applied to improve the loading capacity and allow for a more sustained drug release.

## 3. Materials and Methods

### 3.1. Synthesis of Mesoporous Bioactive Glass Particles

A modified microemulsion-assisted sol–gel synthesis [45,48] with simultaneous application of ultrasound was used to obtain pristine and Sr,Mg-doped MBGNs, denoted as cBAG and SrMgBAG, respectively. Ultrasound application causes acoustic cavitation, the formation, growth, and collapse of bubbles, which produces hot spots that can rapidly initiate the reaction [65].

Hexadecyltrimethylammonium bromide (CTAB) was used as a surfactant and structure-directing agent for obtaining mesoporous structure, while tetraethyl orthosilicate and Ca-, Mg-, and Sr-nitrates were used as silica and cation sources, respectively. Briefly, 7.00 g of CTAB (≥99%, Sigma Aldrich, St. Louis, MO, USA) was dissolved in 330 mL deionized (DI) water at 30 °C, and then 100 mL ethyl acetate (≥99.5%, HPLC grade, Fisher Chemical, Hampton, NH, USA) was slowly added at a speed of 45 ± 5 drop/min. The emulsion formed was stirred for 30 min before simultaneous high-power ultrasound probe was applied in a pulsed regime (10 s on/10 s off) for 30 min. In order to maintain temperature of 30 ± 2 °C, an ice bath was used during the ultrasound application. Three milliliters of aqueous ammonia (28–30 wt.% NH_3_/H_2_O, Acros Organics, Antwerpen, Belgium) was added and stirred for 15 min before TEOS (≥99% (GC), Sigma Aldrich, St. Louis, MO, USA) was added drop-wise at speed of 45 ± 5 drop/min (molar ratio CTAB/TEOS = 1/9). Nominal composition (mol.%) of MBGNs was SiO_2_/CaO/MgO/SrO = 70/30/0/0 for pristine and 70/20/5/5 for Sr,Mg-doped. Corresponding amounts of calcium nitrate tetrahydrate Ca(NO_3_)_2_4H_2_O (>99%, Thermo Scientific, Waltham, MA, USA), magnesium nitrate hexahydrate Mg(NO_3_)_2_6H_2_O (98–102%, Carlo Erba, Cornaredo, Italy), and anhydrous strontium nitrate Sr(NO_3_)_2_ (≥99%, Acros Organics, Antwerpen, Belgium), respectively, were added at 30 min intervals and finally stirred for an additional 4 h. The resulting suspension was centrifuged for 5 min at 10,000 rpm, and washed twice with DI water and once with absolute ethanol. Washed precipitates were dried for 10 h at 60 °C and then calcinated in air at 600 °C for 5 h at a heating rate of 2 °C/min.

### 3.2. Characterization of Obtained Particles

#### 3.2.1. Compositional Analysis

The amorphous structure of bioactive glass particles was evaluated by using X-ray diffraction (XRD) analysis, conducted on a Rigaku Smartlab diffractometer (Rigaku Coorporation, Tokyo, Japan) with CuKα radiation (1.54 Å) in the 2θ angle ranging from 10° to 70° with a scan rate of 0.02° s^−1^.

The composition of the particles obtained was determined by employing ICP-OES analysis after microwave digestion using an Advanced Microwave Digestion System (Ethos 1, Milestone, Sorisole, Italy) with a HPR-1000/10S high pressure segmented rotor. About 50 mg of sample was precisely weighed with accuracy ±0.1 mg and placed in the pressure-resistant PTFE vessel and mixed with of 3 mL H_2_SO_4_ (96 wt.%, Suprapur^®^), 3 mL H_3_PO_4_ (ACS reagent, ≥85 wt.%, Merck, Darmstadt, Germany), 1 mL HNO_3_ (65 wt.%, Suprapur^®,^ Merck, Darmstadt, Germany), and 1 mL HF (40 wt.%, Suprapur^®^) (Merck, Darmstadt, Germany). The temperature was gradually raised with microwave power (0–1000 W): linearity from 25 to 200 °C in the first 15 min, maintained at 200 °C for the next 20 min, and then decreased rapidly to room temperature. After cooling and without filtration, the solution was diluted to a fixed volume (50 mL) in the volumetric flask with ultrapure water. Ultrapure water with a conductivity of 0.05 µS/cm was prepared using a Barnstead™ GenPure™ Pro (Thermo Scientific, Waltham, MA, USA).

ICP-OES measurement: The chemical composition of cBAG and SrMgBAG were determined by inductively coupled plasma optical emission spectrometry, ICP-OES (iCAP 6500 Duo ICP, Thermo Fisher Scientific, Cambridge, UK). The emission lines of the investigated elements were as follows: Ca II 396.847 nm, Mg II 379.553 nm, Sr II 421.552 nm, and Si I 251.611 nm. Quality control was carried out using blank samples, matrix-matched calibration solutions, and triplicate analyses of each sample. The reliability of measurements was approved by a relative standard deviation (RSD) < 1%. The limit of detection (LOD) was in the range of 0.05–1.5 µg/L, and the limit of quantification (LOQ) was in the range of 0.1–5 µg/L in solutions of totally mineralized samples. The analytical process quality control (QC) was performed using the certified reference material (CRM) of EPA Method 200.7 LPC Solution (ULTRA Scientific, Santa Clara, CA, USA). Recovery of measured concentrations with certified values was 98–103%. Concentrations of elements of sample were expressed as mol%.

#### 3.2.2. Morphology and Textural Properties

A field emission scanning electron microscope (FE-SEM, Tescan Mira 3 XMU, Brno, Czech Republic) operated at 20 keV was used to analyze the morphology of the MBGNs. Prior to the analysis, particles were coated with a thin layer of gold using a sputter coater (Polaron SC503, Fisons Instruments, Glasgow, UK). To analyze the mesoporous structure of the MBGNs, a transmission electron microscope (TEM, FEI Talos F200X with an X-FEG source, Hillsboro, OR, USA) operating at 200 kV was used. The micrographs were recorded on a CCD camera with a resolution of 4096 × 4096 pixels using the user interface software package. An energy dispersive X-ray spectroscopy (EDX) system attached to the TEM operating in the scanning transmission (STEM) mode was used for element color mapping. High-angle annular dark-field (HAADF) images were captured in nanoprobe-TEM mode with probes less than 1 nm in size and a camera length of ~200 mm. The samples for TEM were first dispersed into ethanol and then a drop of the solution was placed on a carbon-coated copper grid, which was then air-dried.

The specific surface area (SSA) of the samples was determined by N_2_ adsorption (Micromeritics ASAP 2020, Norcross, GA, USA) and calculated according to the Brunauer, Emmett, and Teller (BET) method from the linear part of the nitrogen adsorption isotherms [66]. The total pore volume (*V_tot_*) was given at *p*/*p*_0_ = 0.998. The volume of the mesopores (*V_meso_*) was calculated according to the Barrett, Joyner, and Halenda (BJH) method from the desorption branch of the isotherm, while the volume of micropores (*V_micro_*) was calculated from an alpha-S plot [67].

The zeta potential (ζ—potential) of the particles was determined by Zetasizer NANO ZS (Malvern Instruments, Malvern, UK) dispersed in two different solvents: ethanol and distilled water. Average particle size was determined by analyzing FESEM micrographs of particles taken at 100,000× magnification with Image-J software 1.53k, where *n* = 200 particles were taken into account.

#### 3.2.3. In Vitro Bioactivity

Bioactivity of glass particles was assessed by immersing previously sterilized particles in Kokubo simulated body fluid (SBF) at ratio of 1 mg/mL for 7 days and 14 days at 37 °C. Tempered and sterilized SBF using 0.22 μm filters was refreshed every 48 h under sterile conditions. Sterilization of the particles was performed by heating at 180 °C for 4 h. At the 7 or 14 days time points, particles were collected by centrifugation, dried, and sputter coated with gold to be analyzed by FE-SEM operated at 20 keV.

#### 3.2.4. Dissolution Extracts Preparation and Ion Release Evaluation

Sterilized particles (3 h at 180 °C) were added at concentration 1 mg/mL to a corresponding volume of Dulbecco’s modified Eagle’s medium-high glucose (DMEM-HG, Gibco, Thermo Fisher Scientific, Inc., Waltham, MA, USA) and Dulbecco’s modified Eagle medium-low glucose (DMEM-LG, Gibco, Thermo Fisher Scientific, Inc. Waltham, MA, USA) without any supplements, and the resulting solutions were placed on an orbital shaker for 24 h and 7 days at 37 °C. The solutions were centrifuged at 5000 rpm for 15 min and then filtered using 0.22 µm filters to evaluate the concentration of calcium, strontium, magnesium, silicon, and phosphorus in DMEM at given time points. For the 0 h time point, nontreated DMEM mediums were used. The ion release profile in cell media was investigated by the previously mentioned ICP-OES measurement. The dissolution extracts obtained were denoted as cBAG LG, SrMgBAG LG, cBAG HG, and SrMgBAG HG according to the DMEM type used.

### 3.3. Assessment of Biological Properties of Mesoporous Bioactive Glass Particles

Indirect biological assays were performed with the cell culture media conditioned with MBGN particles for 24 h at a concentration of 1 mg/mL, in the same way as dissolution extracts were prepared (described in previous section).

#### 3.3.1. Two-Dimensional Experiments with Stem Cells

##### Two-Dimensional Cell Cultures with Human Bone Marrow-Derived Mesenchymal Stem Cells (BM-MSCs)

Human telomerase reverse transcriptase (hTERT)-immortalized BM-MSCs of clonal line Y201 [68] were cultivated in DMEM-LG supplemented with 15% fetal bovine serume (FBS, Gibco, Thermo Fisher Scientific, Inc. Waltham, MA, USA) and 1% antibiotics (penicillin/streptomycin, Gibco, Thermo Fisher Scientific, Inc., Waltham, MA, USA) at 37 °C and 5% CO_2_ atmosphere. Cells were grown until 80–90% confluence, and then collected by enzymatic digestion using trypsin/ethylenediamine tetraacetic acid (trypsin/EDTA, Gibco, Thermo Fisher Scientific, Inc., Waltham, MA, USA) prior to each assay.

##### Cell Seeding

BM-MSCs were seeded at a density of 5000 cells/well on 12-well cell culture plates. Cells were incubated overnight to allow cell adhesion, and the day after, four different media were added: DMEM-LG supplemented with 15% FBS, cBAG-LG and SrMgBAG-LG conditioned media supplemented with 15% FBS, and osteogenic medium (DMEM-LG supplemented with 10% FBS, 20 mM β-glycerophosphate, 50 μM ascorbic acid-2-phosphate, and 10^−7^ M dexamethasone). Samples were cultivated for 7 days, and were evaluated in triplicates (*n* = 3).

##### Resazurin Assay

The resazurin reduction assay is aimed at measuring cellular metabolic activity as an indicator of cell viability and growth. For this purpose, resazurin sodium salt (powder, Merck, Darmstadt, Germany) was used to prepare 1% resazurin solution in 1X phosphate buffered saline (PBS), and at days 1 and 3 of cultivation, 15 µL of 1% resazurin solution was added to the wells containing 1 mL of culture medium to reach a final concentration of 0.015% (*v*/*v*). Following 3–4 h of incubation, fluorescence was quantified using 100 µL aliquots at excitation and emission wavelengths of 530 and 590 nm, respectively, using a microplate reader (Tecan GENios Microplate Reader, Spark, Tecan Trading AG, Männedorf, Switzerland). The results were averaged over three biological replicates (*n* = 3).

##### Alkaline Phosphatase (ALP) Analysis

To detect alkaline phosphatase (ALP) activity, the colorimetric p-nitrophenyl phosphate (pNPP) assay (Alkaline Phosphatase Assay Kit (Colorimetric), Abcam, Cambridge, UK) was performed on cell lysates obtained from cells after 7 days of cultivation. Cell lysates were obtained by discarding the spent media, washing with 1X PBS, adding 50 µL of ALP assay buffer, and performing three freeze–thaw cycles at −80 °C. The resulting cell lysates were then centrifuged for 15 min at 4 °C and 12.5 xg, and the supernatant was collected in 1.5 mL microcentrifuge tubes and stored at −80 °C.

The ALP activity assay was performed according to the manufacturer’s instructions; 50 µL of each cell lysate was mixed with 50 µL of 5 mM pNPP solution. The plate was incubated at 25 °C for 60 min and protected from light; the reaction was stopped by adding 20 µL of stop solution, and the absorbance was measured at 405 nm using a microplate reader (Tecan GENios Microplate Reader, Spark, Tecan Trading AG, Männedorf, Switzerland). Finally, ALP activity was calculated according to the following equation: ALP Activity = (B/∆T × V) × D, where B is the amount of pNP in the sample-well calculated from the obtained standard curve in μmol, ΔT is the reaction time in min, V is the original sample volume added to the reaction-well in mL, and D is the sample dilution factor.

##### Alizarin Red S Staining

To evaluate mineralization via calcium deposition, Alizarin red S (ARS, Merck, Darmstadt, Germany) staining was performed following the manufacturer’s instructions at day 7 of cultivation. Cells were fixed in 4% paraformaldehyde (PFA) at room temperature for 15 min, and then stained using 40 mM ARS solution for 30 min with gentle shaking. The stained monolayers were visualized by phase microscopy using an inverted microscope. For quantification of the staining, 10% acetic acid was used, and the plate was incubated for 30 min with gentle shaking. The cells in 10% acetic acid were scraped and transferred to 1.5 mL microcentrifuge tubes, and then were vortexed for 30 s, heated at 85 °C for 10 min, transferred to ice for 5 min, and centrifuged at 20,000× *g* for 15 min. Finally, 250 µL of the supernatant was moved to a new 1.5 mL microcentrifuge tube, and 100 µL of 10% ammonium hydroxide was added to neutralize the acid. Aliquots of 100 µL were collected in a 96-well plate (opaque-walled, transparent-bottomed plates), and the absorbance was read at 405 nm using a microplate reader (Tecan GENios Microplate Reader, Spark, Tecan Trading AG, Männedorf, Switzerland). The results were averaged over three biological replicates (*n* = 3).

#### 3.3.2. Three-Dimensional Cell Culture Model with Human Endothelial Cells

##### Cell Culture Conditions

The endothelial cell line EA.hy926 was purchased from the American Type Culture Collection (ATCC, Manassas, VA, USA) and cultivated in DMEM-HG supplemented with 10% fetal bovine serum (FBS, Gibco, Thermo Fisher Scientific, Inc.,Waltham, MA, USA) and 1% antibiotics (penicillin/streptomycin, Gibco, Thermo Fisher Scientific, Inc., Waltham, MA, USA) at 37 °C and 5% CO_2_ atmosphere. Cells were grown until 80–90% confluence, and then collected by enzymatic digestion using trypsin/ethylenediamine tetraacetic acid (trypsin/EDTA, Gibco, Thermo Fisher Scientific, Inc., Waltham, MA, USA) prior to each assay.

##### Three-Dimensional Model Preparation

Approximately 150,000 EA.hy926 were suspended in 200 µL of 0.5% collagen (5 mg/mL PureCol EZ Gel solution, Merck, Darmstadt, Germany) and placed in the well of a 24-well cell culture plate. The plate was placed at 37 °C for around 4 h to allow collagen to polymerize, and then four different media were added: DMEM-HG supplemented with 10% FBS; DMEM-HG conditioned with cBAG and supplemented with 10% FBS; DMEM-HG conditioned with SrMgBAG and supplemented with 10% FBS; and DMEM-HG supplemented with 10% FBS and containing 100 ng/mL vascular endothelial growth factor (VEGF). Samples were cultivated for 3 and 7 days, and were tested in triplicates (*n* = 3).

##### Resazurin Reduction Assay

The resazurin reduction assay was performed as previously described on days 1, 3, and 7 of cultivation. The results were averaged over three biological replicates (*n* = 3).

##### Fluorescence Staining

To visualize the morphology and spreading of endothelial cells, the cells were fixed at day 3 and day 7 of cultivation using 4% PFA for 15 min at room temperature, and then the wells were then washed 3× with 1X PBS. The actin filaments in the cytoskeleton and the nuclei of EA.hy926 cells were stained using phalloidin and 4′,6-diamidino-2-phenylindole (DAPI) (Actin Cytoskeleton/Focal Adhesion Staining Kit, Merck, Darmstadt, Germany), respectively, as follows: 0.5X phalloidin in 0.5% bovine serum albumin (BSA) in 1X PBS was added to wells, and after an overnight incubation at 4 °C, the wells were washed 3× with 1X PBS; the cells were then permeabilized using 1X Triton, washed 3× with 1X PBS, stained with 1 μg/mL DAPI in 0.5% BSA in 1X PBS, and then washed 3× with 1X PBS. Representative images were taken using THUNDER Imager Live Cell and 3D Assay (Leica Microsystems, Wetzlar, Germany) at 5× and 20× magnifications.

##### Statistical Analysis

In this study, all experiments were conducted with three biological replicates. Continuous variables were summarized as means ± standard deviation (SD). Statistical comparisons were performed using a one-way analysis of variance (ANOVA), followed by the Tukey post hoc test. Statistical significance was determined based on adjusted *p*-values (<0.05). The analysis was conducted using R [69], RStudio [70], and the Tidyverse package [71].

### 3.4. Drug Loading and Release Evaluation

Ibuprofen (IBU, ≥98%, Sigma Aldrich, St. Louis, MO, USA) was used as a model anti-inflammatory drug. Briefly, 200 mg of cBAG and SrMgBAG were dispersed in 50 mg/mL ethanol solution of IBU for 2 min in an ultrasound bath and stirred for 24 h at room temperature. In vitro drug release of IBU from cBAG and SrMgBAG was performed by the dialysis tubing diffusion method; 100 mg of cBAG@IBU and SrMgBAG@IBU was placed in dialysis tubing (Servapor, MWCO 12,000–14,000, Serva electrophoresis, Heidelberg, Germany) and introduced into a container with 40 mL of PBS (pH = 7.4, 37 °C). At each time point, 2 mL of the release medium was removed, filtered, and assayed by a UV-VIS spectrophotometer (UV-1800, Shimadzu Coorporation, Kyoto, Japan) at wavelength of λ = 264 nm, and the aliquot was returned back to the PBS solution. Thermal analysis (thermogravimetric TGA) of pure particles and particles loaded with IBU before and after 55 h of drug release was performed on an SDT Q-600 simultaneous DSC-TGA instrument (TA Instruments, New Castle, DE, USA). The samples were heated in a standard alumina crucible from room temperature to 500 °C at a heating rate of 10 °C min^−1^ under nitrogen with a flow rate of 0.1 dm^3^ min^−1^. Loading capacity was calculated as the weight loss from 200–500 °C of the samples loaded with IBU determined by the thermal analysis, since no drug was found after 55 h of release.

## 4. Conclusions

Sr,Mg-doped mesoporous bioactive glass nanoparticles were successfully obtained by a microemulsion technique modified by ultrasonic waves. The incorporation of dopant ions led to different mesostructure: radial dendritic compared to worm-like mesopores in cBAG, with a high specific surface area, and increased pore volume compared to pristine cBAG. Despite this, the loading capacity of ibuprofen was somewhat reduced and the drug release rate was slightly accelerated owing to the dendritic pores open at the external surface, thus enabling faster drug diffusion. A faster delivery rate of ibuprofen could provide quicker pain and/or inflammation relief in post-surgical recovery. Additionally, the drug-loading capacity of SrMgBAG could be further improved by surface functionalization strategies and optimization of drug and solvent types, which will be the objective of future investigations.

Indirect biological evaluation assays of cBAG and SrMgBAG particles confirmed that particles are cytocompatible, and have ability to slightly induce BM-MSCs osteogenic potential due to the improvement in ALP production and calcium deposition. Moreover, they have shown to significantly stimulate the viability and proliferation of EA.hy926 cells in 3D culture models. Interestingly, there was no significant difference found between SrMgBAG and cBAG effects on BM-MSCs, while the fluorescence staining revealed more prominent overlapping growths of EA.hy926 cells in the cBAG group after 7 days of cultivation compared to SrMgBAG due to greater cell proliferation, while the SrMgBAG particles had more similar effect to the VEGF group on the cytoskeletal organization of endothelial cells due to the angiogenic Mg^2+^ ions released. In conclusion, Sr,Mg-doped MBGNs can be considered promising multifunctional biomaterials due to their ability to stimulate cell proliferation, osteogenic differentiation, and angiogenesis, and their capacity to locally deliver drugs.

## Figures and Tables

**Table 1:** ICP results of the composition of obtained MBGNs.

Sample	Composition of Bioactive Glass (mol.%)			
	SiO_2_	CaO	MgO	SrO
cBAG	91.61	8.39	/	/
SrMgBAG	88.96	6.04	4.74	0.26

**Table 2:** Textural properties determined by Brunauer–Emmett–Teller (BET) analysis and ζ—potential values of pristine bioactive glass particles (cBAG) and doped with Sr and Mg ions (SrMgBAG). *SSA*—specific surface area, *V_to__t_*—total pore volume; *V_meso_*—volume of mesopores, *V_micro_*—volume of micropores, *D_p_*—average pore size, *D_max_*—pore size of most abundant pores, and *d_avg_*—average particle size.

Sample	*SSA* (m^2^/g)	*V_tot_* (cm^3^/g)	*V_meso_* (cm^3^)	*V_micro_* (cm^3^)	*D_p_* (nm)	*D_max_* (nm)	*d_avg_* (nm) *SEM*	*ζ—Potential* (mV)	
								Ethanol	H_2_O
cBAG	386.17	0.8382	0.801	0.114	7.356	6.376	117.97 ± 14.86	−10	−11.5
SrMgBAG	383.40	0.9235	0.867	0.119	9.530	2.919	112.46 ± 14.72	0.17	−9.49

## Data Availability

All data presented in this study are included in the published article. Further inquiries can be directed to the corresponding author.
